# Editorial: Immunopathology of Chronic Bacterial and Viral Diseases Prevalent in Latin America

**DOI:** 10.3389/fimmu.2020.00749

**Published:** 2020-04-22

**Authors:** Rosana Pelayo, Luis F. García, Leopoldo Santos-Argumedo

**Affiliations:** ^1^Eastern Biomedical Research Center CIBIOR, Mexican Institute for Social Security, Puebla, Mexico; ^2^Group of Cellular Immunology and Immunogenetics, University of Antioquia, Medellín, Colombia; ^3^Department of Molecular Biomedicine, Center for Research and Advanced Studies, National Polytechnic Institute, (CINVESTAV-IPN), CDMX, Mexico City, Mexico

**Keywords:** immunopathology, chronic, bacterial, viral, infections, Latin America

Over the last couple of decades, global burden disease studies have shown a decline in mortality rate leading to an increase in life expectancy and dynamic temporal patterns. Accordingly, Latin America has been experiencing an epidemiological transition process, characterized by decreasing incidences of some infectious and contagious diseases, the improvement in maternal and children survival parameters, and a growing number of chronic degenerative diseases. Despite this, the life expectancy of adults does not yet reach that of high-income countries and the epidemiological transition has been heterogeneous in the region, with some countries exhibiting favorable health indicators resulting from better-developed economy and good public policies, while other countries suffering from communicable diseases that relates to poverty, inadequate sanitation, close contact with infectious vectors and deficient access to health services. Such “neglected infectious diseases” show a substantial disease burden in Latin America and include Dengue, with increased incidences in the last decade. Moreover, the emergence of new pathologies related to arboviruses, such as Zika and Chikungunya, has added to the complexity of the problem and continue to be a center of scientific discussion with regard to regional immunology.

From May 14–18, 2018, the Latin American Association of Immunology (ALAI) and the Mexican Society of Immunology (SMI) co-organized their XII Congress and XXIII Congress, respectively, which featured an outstanding program in basic, translational and clinical immunology. The complete work, including one pre-congress cytometry meeting, 10 plenary lectures, 23 symposia, 18 workshops and 484 poster presentations, was published within a *Frontiers Abstract Book* (https://www.frontiersin.org/books/Immuno_Mexico_2018_XII_Congress_of_the_Latin_American_Association_of_Immunology_and_XXIII_Congress_/1637). Moreover, with the support of the International Union of Immunology Societies (IUIS), this Research Topic was launched, devoted to current and in-progress scientific knowledge on basic immunopathological aspects of chronic infectious diseases and their control in our region.

“Immunopathology of Chronic Bacterial and Viral Diseases Prevalent in Latin America” includes 12 Original Research articles, 5 Reviews, 2 Mini-reviews and one Case Report, providing a comprehensive overview of the advancements in some of the pathogenic agents that have been the cause of emerging and re-emerging diseases in Latin America, such as bacterial pathogens: *Salmonella enteritidis*; *Salmonella typhimurium*; *Salmonella typhi*; *Brucella abortus;* and *Klebsiella pneumoniae*, and viral pathogens: human immunodeficiency virus (HIV); dengue; zika; chikungunya; human papillomavirus (HPV); and Epstein-Barr virus (EBV). As substantial efforts to assemble the bridge between basic research and clinical applications are presented, we hope this Research Topic contributes as one of the multilateral actions that benefit regional science, education and health.

A series of four publications are dedicated to *Brucella* spp., a zoonoses transmitted to humans through consumption of contaminated products, representing a health and financial problem in livestock areas. Given that the infection is mainly acquired by ingestion or inhalation of bacteria, López-Santiago et al. review the role of mucosal immune responses. In the gastrointestinal tract, *Brucella* spp. are able to neutralize the effects of gastric juice and bile salts and apparently uses epithelial M cells to enter the mucosa without inflammation. In that respect, Pasquevich et al. propose that the Omp19 outer membrane lipoprotein of *Brucella abortus*, endowed with proteolytic activity, may help this microorganism to evade destruction by the gastrointestinal proteases. Deficiency of Omp19 results in a lower infective ability when administered orally in susceptible mice. Furthermore, using a murine model of *Brucella abortus* infection, Gutiérrez-Jiménez et al. show that polymorphonuclear cells (PMNs) capable of phagocytizing bacteria, display membrane phosphatidylserine and are phagocytized by macrophages that secrete high levels of IL-10 and low levels of TNFα. Bacterial replication is higher in macrophages that ingest dying-infected PMNs, suggesting a “Trojan horse” strategy for its dissemination. Finally, *Brucella abortus* has the ability to interfere with protective immune responses through various mechanisms that include the disruption of pathogen recognition receptor signaling. Here, Milillo et al. report the contribution of RNA from *Brucella* to a specific decrease in MHC class II molecules, and without interference with interferon-gamma mediated manifestations.

Among bacterial infectious diseases, typhoid fever is also a public health concern in Latin America as a leading cause of invasive infections that show increasing drug resistance in children. Recent work on *Salmonella* porins has resulted in potential diagnostic tools and vaccine candidates. Valero-Pacheco et al. report a bioinformatical analysis of the OmpC porin in 8 types of thyphoidal and non-thyphoidal *Salmonella*. Their results show several highly conserved amino acid sequences in the transmembrane β in β-barrel, harboring MHC-class II restricted epitopes that may function as vaccine candidates. In an interesting twist, Mateos-Chávez et al. report an engineered attenuated enteric *Salmonella* mutant, capable of delivering antitumor peptides by using its secretion mechanisms. Notably, the intravenous administration of a modified *Salmonella*, harboring a peptide of the pro-apoptotic protein Bax, reduces the tumor activity in a murine xenograft model with “Ramos” B lymphoma cells.

*Klebsiella pneumoniae* SP258 is a hyper-endemic clone resistant to carbapenem and responsible for common severe infections in intensive care units. Castillo et al. compare the capacity of *K. pneumoniae* SP258 to other *K. pneumoniae* strains and *Escherichia coli*, of affecting PMN responses. Although both bacteria were similarly phagocytized, *K. pneumoniae* SP258 does not induce the production of reactive oxygen species (ROS) or NETosis, while *E. coli* does. Moreover, LPS from *K. pneumoniae* mediates the inhibition of PMNs responses, and SP258 apparently uses this mechanism to evade the innate immune response.

A number of viral infections are also discussed from the immunopathological perspective. An interesting Mini-Review from Reyes-Sandoval and Ludert, assembles a wealth of information relevant to the biology of non-structural proteins of the Dengue and Zika arboviruses and the cross-reaction of anti-NS1 antibodies with host cells, which potentially weakens its use as a therapeutic target. In addition, Arévalo Romero et al. describe the potential transmission of Zika virus through vector-independent mechanisms. The authors provide an analysis of the impact and consequences of the sexual transmission of Zika virus on disease dynamics. Special mention is made on the long viral persistence in male gonads, a site recognized as immune-privileged, making men potential reservoirs for infection in non-endemic areas. Original research from Shrivastava et al. addresses central mechanisms contributing to pro-inflammatory immunopathogenesis in dengue viral infection. They show the capability of DENV-2 NS2A and NS2B proteins of inducing IL-1β, a process mediated by NLRP3 inflammasome activation in endothelial cells and directly related to calcium mobilization. Of note, Fernandes et al. report a case of severe Chikungunya fever and vesiculobullous lesions treated with immunoglobulin. The 5-day treatment with intravenous immunoglobulin achieved a total recovery of the patient's lesions over 10 days, with no clinical signs of the disease at discharge. This adjunctive therapy may ameliorate severe cases of Chikungunya fever.

Junin virus is the etiological agent of Argentine hemorrhagic fever. Ferrer et al. compare the effect of infection in human monocyte-derived macrophages with attenuated and virulent strains of this arenavirus. Their results show that while the attenuated strain promotes classically activated macrophages, the virulent strain infection results in alternatively activated cells. A skew in macrophage polarization induced by Junin virus infection is explained by the increased expression of MERKT receptor, SOCS1, and SOCS3, during virulent-strain infection.

HIV is still a challenge, due to a lack of vaccination strategies, the cumbersome budgetary burden of antiviral drugs, and the poor prognosis with tuberculosis co-infection. Here, Perdomo-Celis et al. describe the regenerative effects of current HIV antiretroviral drugs on the immune system, emphasizing the promising role of CD8 T cell subpopulations in the immunological reconstitution during treatment. A number of strategies to promote CD8 T function are suggested to rapidly transform the burden. Moreover, Alvarez et al. discuss the role of Vitamin D in HIV infection. Worth noting, the review provides a comprehensive overview of the many clinical studies showing the beneficial functions of this hormone in immune cell regulation and its potential use as a protective nutritional supplement. Meanwhile, Giacoia-Gripp et al. evaluate the changes in the profile of circulating innate lymphocytes in patients coinfected with HIV and tuberculosis (TB), with or without IRIS during antiretroviral therapy, compared to patients with only HIV or TB infection and healthy controls. HIV/TB patients show high numbers of circulating γδ ted δ^+^/Vδ^−^ ratio and increased expression of CD158a, NKp80, and NKG2C on NK cells in HIV/TB IRIS+ compared to coinfected patients without IRIS. Finally, the association of *Candida* spp. infections with antiretroviral treatment in clinical periodontitis is controversial. In their study, Lomeli-Martinez et al. show a potential association in the abundance and the diversity of *Candida* spp. with low numbers of CD4+ T cells and the use of antiretroviral drugs. The most abundant species was *C. albicans*, followed by *C. glabrata, C. tropicalis, C. krusei*, and *C. dubliniensis*.

Influenza virus evolves by either antigenic drift that is responsible for seasonal variability or an antigenic shift that is responsible for the emergence of new strains. These phenomena represent tremendous challenges for the development of effective vaccines and show the importance of a continuous analysis of the antibody response elicited by emerging viral epitopes. Padilla-Quirarte et al. extensively review a role of antibodies in protection against influenza virus. They describe the protective mechanisms of antibodies against the different parts of the haemagglutinin and neuramidase molecules present on the lipid membrane. The authors also address the controversial protective role of antibodies directed against viral internal antigens. On the other hand, the family Caliciviridae comprises human noroviruses and sapoviruses, the main etiological agents of acute gastroenteritis. Peñaflor-Téllez et al. review the capacity of Caliciviruses to modulate the immune response. They detail the evasion mechanisms used by Caliciviruses that include inhibition of type I and III interferon gene translation and impairment of antigen presentation, among other evasion mechanisms.

The oncogenic/oncopromotor role of a number of pathogenic viruses has led to unprecedented advances in medical oncology. Accordingly, the HPV-cervical cancer etiological relationship is clear, and in this volume, Artaza-Irigaray et al. demonstrate overexpression of IL-6 in cervical cancer cell lines compared to normal keratinocytes and cervical intraepithelial neoplasia grade 1. Transfection of the normal keratinocyte line HaCaT with E5, E6, and E7 proteins from high and low risk HPV show that E6 protein induces expression and secretion of IL-6 in a p53-independent way. The authors discuss whether IL-6 contributes to a pro-inflammatory and highly proliferative microenvironment leading to cervical tumorigenesis.

The present collection also includes work characterizing tick saliva, where components that affect the host's immune response have been identified. This is an area of study that is gaining recognition as an important factor in the establishment of infections. While insects are generally considered only pathogen vehicles, recent years have witnessed the scientific interest for several biological molecules within their saliva facilitating pathogen infections. Maldonado-Ruiz et al. describe the protein components of tick saliva from native *Amblyomma americanum* species compared to the same species grown in the laboratory. The results show interesting differences in saliva composition, and their relevance is discussed.

Collectively, manuscripts included in this Research Topic highlight the ongoing studies on some of the infectious diseases of health priority in our region. Although far from a comprehensive analysis of Latin American bio-epidemiological complexity, this collection illustrates the complementary basic and applied science that is being conducted by the local immunology community. We hope this multidisciplinary effort helps to inspire young scientists to become committed to deep understanding, prevention and control of regional health problems, and authorities to support new comprehensive strategies and science-based public policies. Clearly, Latin American immunology displays strength and maturity. The current pandemic of COVID-19 caused by SARS-CoV-2 provides a dramatic illustration of the importance of local expertise in the fight against this and future epidemic threats through international cooperation.

## Author Contributions

All authors listed have made a substantial, direct and intellectual contribution to the work, and approved it for publication.

## Conflict of Interest

The authors declare that the research was conducted in the absence of any commercial or financial relationships that could be construed as a potential conflict of interest.

